# Erastin-induced multi-pathway cell death in endometriosis: a mechanistic and translational narrative review

**DOI:** 10.3389/fmed.2025.1594702

**Published:** 2025-09-10

**Authors:** Zhe Gao, Juan Du

**Affiliations:** School of Medical and Life Sciences, Chengdu University of Traditional Chinese Medicine, Chengdu, China

**Keywords:** Erastin, endometriosis, ferroptosis, mitophagy, necroptosis

## Abstract

This narrative review examines the therapeutic potential of Erastin and its derivatives for endometriosis (EMS) by integrating mechanistic, preclinical, and translational perspectives. We conducted a focused review of literature from PubMed and Web of Science Core Collection (WoSCC) through August 2025; following a systematic screening and de-duplication process, 91 studies were included for synthesis. The evidence indicates that within the iron-rich, ROS-prone microenvironment of EMS, Erastin inhibits the system Xc^−^ transporter, depletes intracellular glutathione (GSH), and inactivates GPX4, thereby driving ferroptosis in ectopic endometrial stromal cells. This process engages a coordinated network of regulated cell death that extends beyond ferroptosis to include crosstalk with necroptosis and pyroptosis, while being critically modulated by ferritinophagy and the paradoxical role of defective mitophagy. Despite the development of next-generation analogs with improved pharmacological properties, clinical translation is constrained by a narrow therapeutic window due to on-target and off-target toxicities. To overcome these limitations, we propose that future strategies must prioritize lesion-focused drug delivery, such as nanocarriers and triggerable prodrugs, alongside biomarker-guided treatment regimens to decouple efficacy from systemic risk, paving a credible path for the clinical application of Erastin-class agents in EMS.

## Graphical abstract

Erastin-induced cell death pathways in endometriosis. 
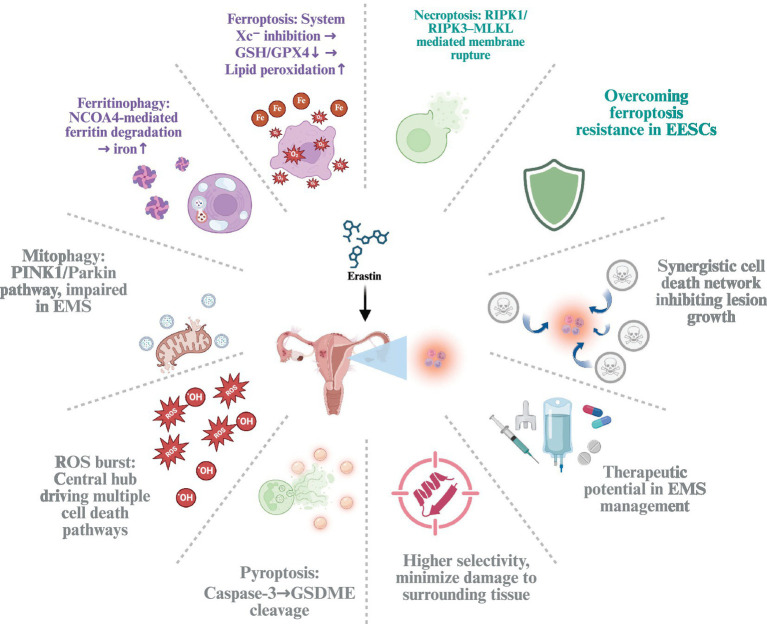

## 1 Introduction

Affecting an estimated 6–10% of women of reproductive age, endometriosis (EMS) is a common gynecological disorder that significantly impacts physical and mental health, often manifesting as a debilitating clinical syndrome of chronic pelvic pain, severe dysmenorrhea, dyspareunia, and associated infertility (1). This disease is characterized by the presence of endometrial tissue, which should normally reside within the uterine cavity, appearing in locations outside the uterus where it invades and proliferates, commonly involving the ovaries and peritoneum (2). The most widely accepted theory for its pathogenesis is retrograde menstruation, where endometrial cells flow backward through the fallopian tubes into the pelvic cavity during menstruation (3). Surgical treatment continues to play a critical role in the management of EMS, especially in cases where symptoms are severe or other treatments have been ineffective. Surgery is suitable for patients who cannot tolerate or do not respond to medical therapies, particularly those with acute pain events, deep EMS, or ovarian endometriomas (4). Additionally, surgery can be used to improve the reproductive environment in patients with EMS-related infertility. In cases of deep EMS, the primary goal of surgery is to alleviate pain and organ obstruction. Clinically, surgical options include laparoscopy and the excision of extrauterine lesions. While laparoscopy is a minimally invasive procedure, its recurrence rate is high, whereas radical surgery, which involves the removal of both ovaries along with the lesions, offers a more definitive approach.

Medical therapies commonly include progestins, GnRH agonists, and oral contraceptives, which can alleviate symptoms to some extent but often cause significant side effects and tend to relapse once the medication is discontinued. The hormonal treatment of EMS is primarily designed to target the endocrine mechanisms underlying the disease and includes several major drug classes. Gonadotropin releasing hormone agonists, such as goserelin, are employed to induce a hypoestrogenic state by initially stimulating and then downregulating pituitary hormone secretion, whereas gonadotropin releasing hormone antagonists, directly block receptor binding for a more rapid onset of action (5). Progestins, such as dienogest, are considered a first-line treatment option, and compared to combined oral contraceptives, oral progestins usually have a better safety and tolerability profile (6). In addition, second-line treatments such as GnRH agonists effectively reduce pain but are compromised by significant menopausal side effects and challenges with long-term tolerability, which has prompted the development of novel oral GnRH antagonists like elagolix, linzagolix, and relugolix that offer dose-dependent estrogen suppression and rapid reversibility. These newer agents enable a more personalized balance between efficacy and safety through strategies such as add-back therapy to mitigate bone mineral density loss and vasomotor symptoms (7, 8). Overall, both surgical and medical treatments face limitations such as high recurrence rates or pronounced adverse effects, and there remains a lack of ideal long-term therapeutic options in clinical practice (9).

In recent years, studies have revealed that cell death plays a critical role in EMS lesions. These lesions frequently exhibit iron overload, oxidative stress, and inflammation, all of which can induce and exacerbate ferroptosis, thereby inhibiting the growth of lesion cells (10). Erastin, as a small-molecule ferroptosis inducer, works by inhibiting the system Xc^−^ transporter-an amino acid antiporter responsible for exchanging extracellular cystine for intracellular glutamate (11)-leading to depletion of intracellular glutathione (GSH), indirectly inactivating glutathione peroxidase 4 (GPX4), and ultimately triggering ferroptosis (12). However, as research has progressed, Erastin’s induction of cell death is not limited solely to ferroptosis but may involve the combined action of multiple cell death pathways (13, 14). Based on the current evidence, we propose that the iron-overloaded, ROS-prone milieu of EMS renders EESCs susceptible to Erastin-triggered, multi-pathway regulated cell death that integrates ferroptosis with necroptotic and pyroptotic signaling, while ferritin handling and mitophagy act as key modulators of susceptibility. In turn, this coordinated engagement of death programs—together with precedent from oncology—supports the view that Erastin has therapeutic potential for EMS when exposure is directed preferentially to lesions and minimized in normal tissues.

## 2 Study selection and literature scope

**Figure 1:**
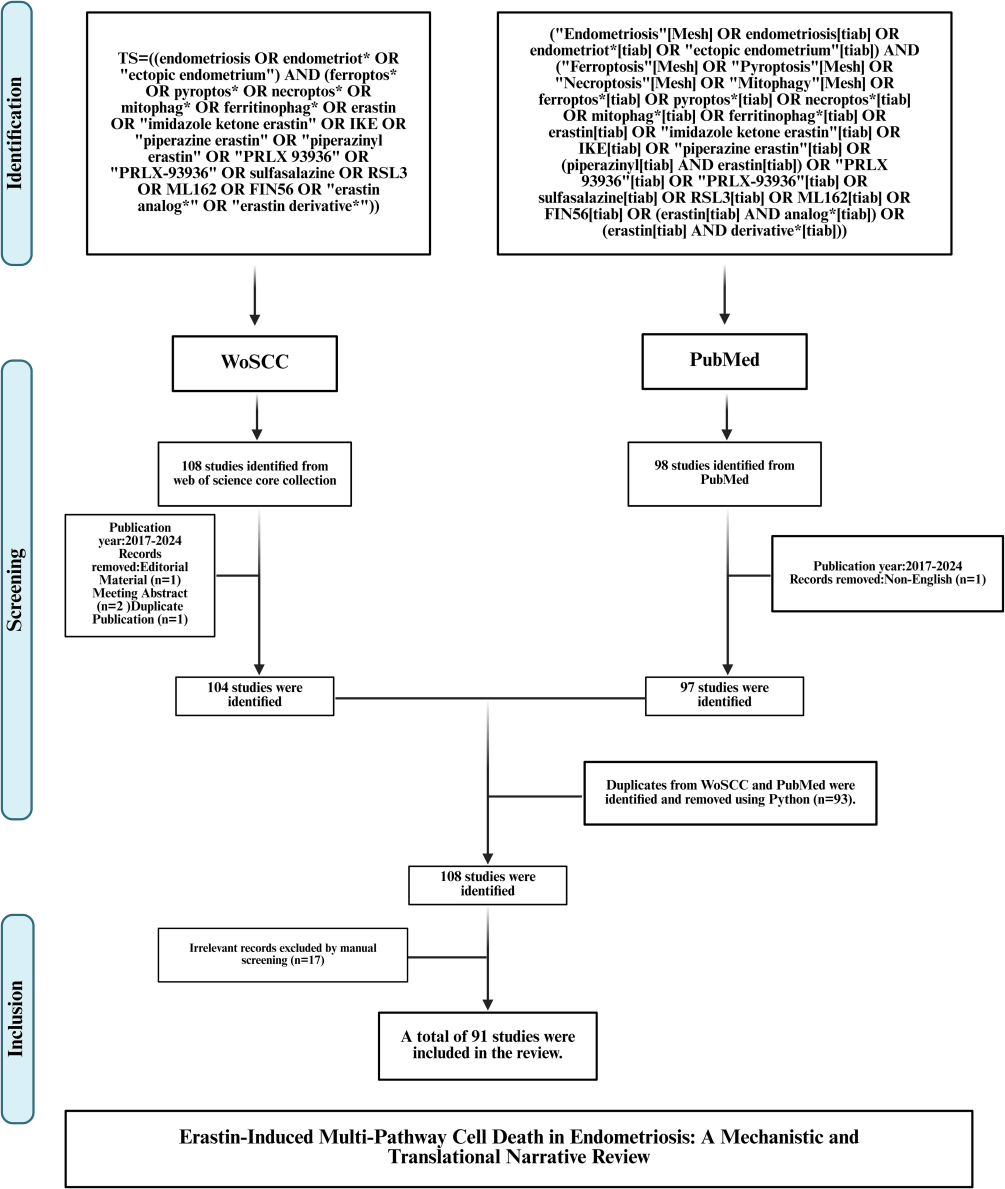
PRISMA 2020 flow diagram of study identification and selection in the PubMed and WoSCC searches.

We conducted a focused narrative review to synthesize published evidence on Erastin and ferroptosis-centered cell-death pathways in endometriosis. Two databases were queried: PubMed and the Web of Science Core Collection (WoSCC). Searches covered each database from inception through August 14, 2025. Database-specific queries were constructed around three concept blocks—endometriosis, regulated cell-death modalities, and Erastin with its analogs—and executed according to each platform’s advanced search rules. The PubMed search returned 98 records; after excluding the single non-English item, 97 records proceeded to screening. The WoSCC search returned 108 records; after removing two conference abstracts, one editorial, and one duplicate, 104 records proceeded to screening. We exported all records with full bibliographic fields and used Python scripts to identify duplicates across sources; ninety-three cross-database duplicates were detected, yielding a combined corpus of 108 unique records for assessment. Two reviewers then independently screened titles, abstracts, and full texts against prespecified criteria, resolving disagreements by consensus. Studies were eligible if they reported published data in English relevant to endometriosis and at least one of the targeted cell-death modalities, with specific attention to Erastin or its analogs in in-vitro, ex-vivo, in-vivo, or human observational contexts. Items not meeting these criteria, including conference materials, editorial matter, letters without primary data, retractions, and topic-irrelevant reports, were excluded. Manual screening of the 108 unique records led to the exclusion of 17 manuscripts for irrelevance, resulting in 91 studies included in the review. To ensure completeness, we also performed forward citation tracking of included articles and examined reference lists to capture additional publications of contextual relevance. Figure 1 shows the literature identification and selection workflow for this narrative review.

## 3 Endometriosis microenvironment: iron–redox dysregulation and cell-death priming

Most of the iron in the human body is contained within circulating red blood cells, where it plays pivotal roles in oxygen transport, metabolic reactions, and DNA synthesis (15). Studies have shown that women with endometriosis have higher levels of iron, ferritin, and hemoglobin in their peritoneal fluid compared to healthy controls (16). Large amounts of free iron are also found around the ovaries infiltrated by ectopic endometrial tissue, and the nearby follicles are overloaded with iron (17). This situation severely impairs oocyte development and quality, potentially contributing to the infertility commonly associated with endometriosis. The initial cause of iron overload in ectopic endometriotic lesions, as well as in peritoneal and follicular fluid, remains unclear, but it may be related to retrograde menstruation and repeated local bleeding of lesions, leading to the excessive degradation and influx of red blood cells. According to Lousse et al., retrograde menstruation and bleeding from ectopic endometrial lesions can transport menstrual endometrial tissue and red blood cells into the peritoneal cavity (18). Some of these tissues and cells are subsequently engulfed, absorbed, and degraded by peritoneal macrophages, storing iron in the form of hemosiderin. In addition, ferritin and hemoglobin are released into the peritoneal fluid. Hemoglobin breakdown releases heme, which is metabolized by heme oxygenase to produce active iron and form iron–ferritin deposits. This process disrupts iron homeostasis, and because the iron-clearing system cannot effectively eliminate the excess iron, an iron-overloaded environment ultimately develops in the peritoneal fluid and endometriotic lesions (19). Moreover, the reflux of menstrual blood into the ovaries and repeated local bleeding in ovarian lesions can create iron overload in the follicular fluid as well. Excessive peritoneal iron accumulation leads to overproduction of reactive oxygen species (ROS)-a group of highly reactive chemicals formed from oxygen, which at high concentrations can damage DNA, RNA, proteins, and lipids within the cell (20)-and enhanced activation of nuclear factor kappa-B (NF-κB), which, by promoting the expression of matrix metalloproteinases (MMPs), exacerbates inflammation, angiogenesis, and cell adhesion. These changes drive the progression of endometriotic lesions and facilitate the development of endometriosis (21). Endometriosis is closely linked to iron overload, and these findings indicate that ectopic endometrial tissue, through repeated bleeding during its invasive growth, evolves into an iron-overloaded state under the influence of macrophages.

**Figure 2:**
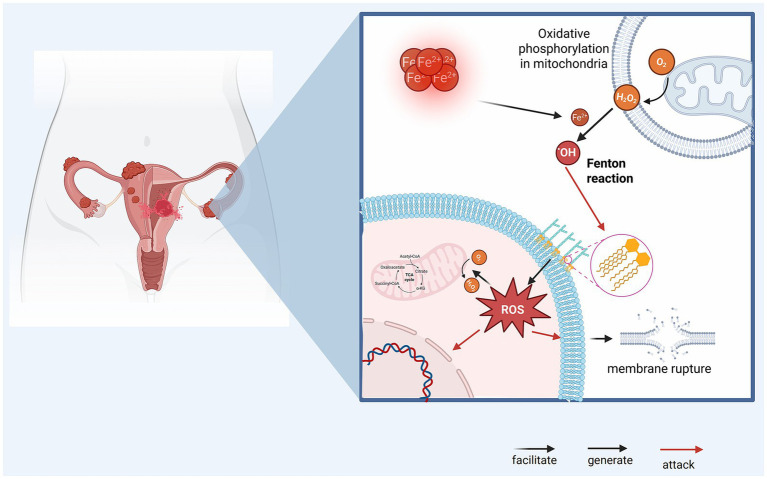
The mechanism of ferroptosis in EMS tissue. The iron-rich microenvironment of endometriotic lesions provides abundant Fe^2+^, which reacts with H₂O₂ via the Fenton reaction to produce highly reactive hydroxyl radicals (•OH). These reactive oxygen species (ROS) then induce lipid peroxidation of the cell membrane, compromising its integrity and ultimately leading to membrane rupture and cell death.

Ferroptosis is a form of programmed cell death characterized by lipid peroxidation, driven by iron and ROS, and was first proposed by Dixon in 2012 (22). In EMS tissue, excessive iron promotes the generation of large amounts of ROS through the Fenton reaction, thereby disturbing the antioxidant equilibrium and triggering oxidative stress. The Fenton reaction is an oxidative process in which ferrous ions (Fe^2+^) catalyze the decomposition of hydrogen peroxide (H₂O₂) into hydroxyl radicals (•OH) (23). Accumulated free iron provides abundant substrate for this reaction, as the iron reacts with hydrogen peroxide to produce highly reactive ROS. These ROS exert substantial oxidative pressure on surrounding cellular structures, particularly on cell membranes rich in polyunsaturated fatty acids (PUFAs) (24). By attacking the double bonds in PUFAs, ROS lead to lipid peroxidation and the formation of lipid peroxides. In the presence of iron, this lipid peroxidation process escalates further, producing large quantities of lipid peroxides that seriously compromise membrane integrity, gradually damaging membrane structure and function (25). As lipid peroxidation intensifies, membrane permeability is altered, eventually culminating in membrane rupture or functional loss and, ultimately, ferroptosis. Figure 2 shows the central mechanism of iron-driven ferroptosis in EMS tissue, where excess iron catalyzes the Fenton reaction to produce ROS, which in turn causes lipid peroxidation and membrane rupture. This iron-overloaded and pro-oxidative milieu creates conditions conducive to ferroptosis in ectopic endometrial tissue in endometriosis. This destructive oxidative process is considered a central mechanism of ferroptosis, iron overload accelerates the production of ROS via the Fenton reaction, which in turn amplifies lipid peroxidation, compromising the cell membrane and leading to ferroptosis.

Resistance to ferroptosis in ectopic endometrial tissue is a crucial feature in the pathogenesis of endometriosis. Although the iron-overloaded microenvironment can destroy surrounding tissue via ferroptosis and induce inflammatory responses (26), its cytotoxic effects on ectopic endometrial tissue are limited, primarily due to the tissue’s ferroptosis resistance (10). The precise mechanisms underlying this resistance remain unclear. Some studies have reported that, in patients with EMS, both normal endometrial stromal cells (NESCs) and ectopic endometrial stromal cells (EESCs) exhibit significantly increased Fibulin-1 expression, which confers ferroptosis resistance on EESCs by inhibiting ferroptosis (27). Additionally, Li et al. discovered that EESCs can proliferate in an iron-rich environment and resist ferroptosis through the ATF4–xCT pathway (28). Furthermore, other research indicates that ferroptosis in EESCs promotes the production of vascular endothelial growth factor A (VEGFA) and interleukin-8 (IL8). Consequently, EESCs secrete angiogenic factors in a paracrine fashion, thereby fueling neovascularization (29) and further advancing endometriosis pathogenesis. This ferroptosis resistance in ectopic endometrial tissue contributes to the difficulty in eradicating lesions and the ongoing progression of the disease. Given this intrinsic resistance to ferroptosis, therapeutic strategies have been developed to pharmacologically bypass these cellular defenses. Among the most studied agents for this purpose is Erastin, a compound that induces ferroptosis through a distinct mechanism.

To counteract the damaging effects of lipid peroxidation, cells have evolved a primary defense system centered on the GSH and GPX4 axis (30). Erastin can directly affect cystine uptake through voltage-dependent anion channels (VDAC) (31). System Xc^−^ is an amino acid exchange system that transports extracellular cystine into the cell while exporting intracellular glutamate (32). Cystine serves as the precursor for GSH, which is a critical intracellular antioxidant (33). By inhibiting system Xc^−^, Erastin reduces cystine uptake, subsequently lowering GSH levels. GSH functions as a cofactor for GPX4, an enzyme that protects cell membranes from oxidative damage by converting lipid peroxides into less harmful lipid alcohols (34). When GSH is depleted, GPX4 loses its ability to neutralize lipid peroxides. Once GPX4 is inactivated, lipid peroxides accumulate within the cell, compromising membrane integrity and eventually causing membrane rupture and cell death (35, 36). Meanwhile, intracellular Fe^2+^ further enhances the lipid peroxidation process via the Fenton reaction, generating additional ROS and promoting ferroptosis. Moreover, Erastin can increase intracellular iron availability by upregulating heme oxygenase, which releases iron by degrading heme (37). Obviously, this ferroptosis mechanism induced by Erastin is clearly distinct from the iron overload–driven ferroptosis observed in EMS tissues, both are based on ROS-induced lipid peroxidation and its damage to the cell membrane, leading to cell death, but the driving mechanisms differ.

Despite the inherent resistance of EMS tissue to ferroptosis, studies have shown that Erastin can still effectively induce ferroptosis in EMS lesions, leading to tissue atrophy and cell death. Li et al. reported in a mouse model of endometriosis that ferroptosis induced by an Erastin analog significantly inhibited the formation of ectopic endometrial lesions and caused a marked atrophy of EESCs. In contrast, iron supplementation had no significant impact on lesion progression in this model, likely due to the intrinsic resistance of EMS lesions to iron overload-induced ferroptosis. Moreover, they found that overexpression of ferroportin (FPN) suppressed Erastin-induced ferroptosis in EESCs, whereas FPN deficiency accelerated this process (38). FPN is the only known transmembrane iron exporter in vertebrates, primarily expressed in duodenal enterocytes, hepatocytes, and macrophages, where it plays a crucial role in maintaining cellular and systemic iron homeostasis (39). Based on these findings, we hypothesize that the ability of Erastin to bypass the ferroptosis resistance of EMS tissue and effectively induce ferroptosis and lesion atrophy may be attributed to its unique ferroptosis-inducing mechanism and its multifaceted regulation of iron homeostasis.

## 4 Necroptosis and pyroptosis in ectopic endometrial stromal cells

Necroptosis is a unique form of programmed cell death that exhibits the morphological characteristics of necrosis while being strictly regulated by specific molecular pathways. Crucially, unlike apoptosis, which is typically immunologically silent, necroptosis results in cell lysis and the release of damage-associated molecular patterns, thereby provoking a potent inflammatory response (40). Unlike apoptosis, its execution is independent of caspases and is instead driven by receptor-interacting protein kinase 1 (RIPK1) and receptor-interacting protein kinase 3 (RIPK3), with mixed lineage kinase domain-like protein (MLKL) acting as the executioner by oligomerizing and forming pores in the plasma membrane, leading to its rupture (41). Recent evidence suggests that necroptosis is already an active process within endometriotic lesions, contributing to the chronic inflammatory environment of the disease (42). Although Erastin is best known as an inducer of ferroptosis, Yu et al. discovered that Erastin can also trigger necroptosis (13). Necroptosis is closely associated with ROS production, and both processes form a positive feedback loop through this shared signaling mechanism. On one hand, ROS generated by NADPH oxidase 1 (NOX1) and mitochondria can activate RIPK1 and further recruit RIPK3 to initiate necroptosis. On the other hand, RIPK3 enhances aerobic respiration by modulating metabolic pathways, thereby increasing ROS production and reinforcing the feedback loop (43).

**Figure 3:**
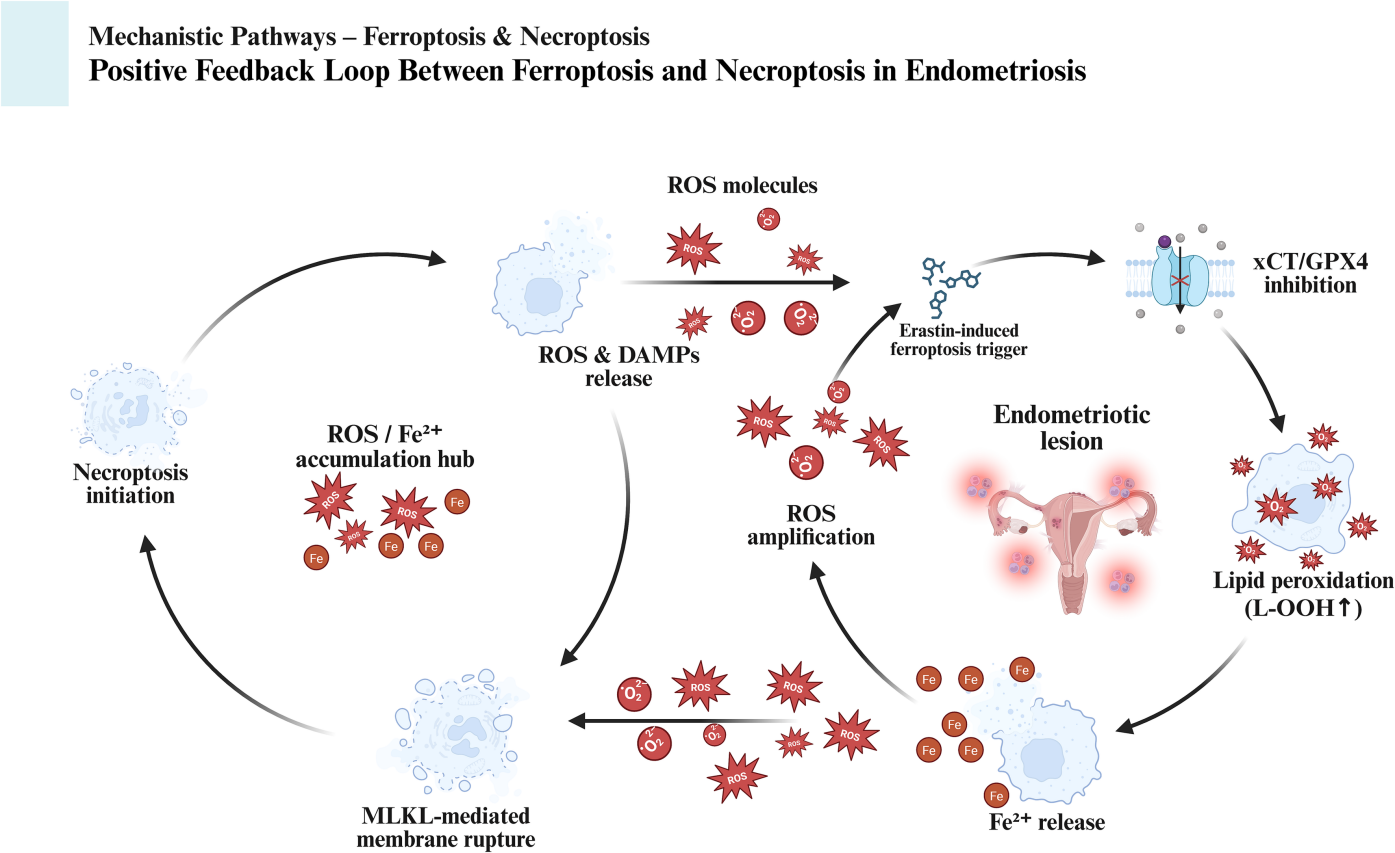
The positive feedback loop of necroptosis induced by Erastin synergizing with ferroptosis. Erastin-triggered ferroptosis leads to plasma membrane rupture and the release of ROS and other DAMPs. This ROS burst activates the RIPK1-RIPK3-MLKL necroptosis pathway. In turn, necroptosis further enhances ROS production, reinforcing the feedback loop and promoting widespread cell death.

Moreover, Erastin-induced ferroptosis is accompanied by plasma membrane rupture and the release of large amounts of ROS. While macrophages and other immune cells can partially clear these ROS, the excessive ROS burden surpasses the clearance capacity of immune cells, allowing residual ROS to further activate necroptosis through positive feedback mechanisms. Therefore, Erastin not only directly induces necroptosis but also amplifies its occurrence through the ROS generated during ferroptosis, establishing a synergistic interaction between ferroptosis and necroptosis. Figure 3 shows the synergistic positive feedback loop between ferroptosis and necroptosis, where ROS released during Erastin-induced ferroptosis triggers necroptosis, which in turn releases more ROS and iron to amplify the overall cell death signal. Based on these findings, we propose that Erastin-induced cell death in EMS tissue is not solely limited to ferroptosis but is also accompanied by necroptosis, further elucidating the complex interplay between these cell death pathways in EMS pathophysiology.

Pyroptosis is a pro-inflammatory, regulated necrotic-like form of cell death. Its signaling cascade is centered around human Caspase-1, Caspase-4, and Caspase-5, or murine Caspase-11 (44). Like necroptosis, pyroptosis is increasingly recognized as a key driver of inflammation in endometriosis, with evidence pointing to the activation of gasdermin proteins in endometriotic tissues (45). Although the exact link between Erastin and pyroptosis remains unclear, Erastin-induced ferroptosis generates a substantial amount of ROS. As a key stress signal, ROS oxidizes the mitochondrial outer membrane protein Tom20, altering its structure and enhancing its ability to recruit Bax. Bax oligomerizes in the mitochondrial outer membrane, forming channels that facilitate the release of cytochrome c into the cytoplasm, subsequently activating Caspase-9, which then triggers Caspase-3 activation through a caspase cascade. Activated Caspase-3 cleaves gasdermin E (GSDME), releasing its N-terminal pore-forming domain (PFD) from the C-terminal inhibitory structure, thereby forming membrane pores and inducing GSDME-mediated pyroptosis (46). Zhou et al. demonstrated that Tom20 or Bax knockout effectively blocks ROS-induced pyroptosis, while antioxidant treatment significantly reduces Tom20 oxidation, Bax mitochondrial recruitment, and GSDME cleavage, further confirming that ROS serves as the central regulator of this signaling pathway (47). Therefore, we hypothesize that pyroptosis may also be involved in Erastin-induced regulated cell death (RCD) in EMS stromal cells. Moreover, the secondary ROS release following cell rupture may further influence ferroptosis and pyroptosis, forming a feedback loop that amplifies cell death progression.

## 5 Selective autophagy and ferritinophagy in lesion redox control

Autophagy is a critical cellular quality control process with a complex, double-edged role in cell fate: it can promote survival by recycling damaged components or contribute to cell death under specific forms of stress. Autophagy is an intracellular degradation process responsible for eliminating proteins, misfolded or damaged organelles, and aged cellular components. During this process, membranes enclose portions of the cytoplasm and targeted cellular components, forming autophagosomes (48). As a classical ferroptosis inducer, Erastin has been found to increase autophagic flux in target cells (14). Indeed, multiple lines of evidence confirm this effect. For instance, Gao et al. demonstrated that Erastin treatment in fibroblasts and fibrosarcoma cells robustly induced the conversion of LC3I to LC3II and the formation of GFP-LC3 puncta, which are definitive hallmarks of autophagy activation (49). Similarly, Li et al. reported that in breast cancer cells, Erastin significantly increased the expression of key autophagy-associated proteins, including beclin-1, ATG5, ATG12, and LC3B, while concurrently decreasing levels of the autophagy substrate p62 (50). Furthermore, Erastin-induced ferroptosis in target cells is accompanied by membrane rupture and the release of large amounts of ROS, which may further enhance autophagy via ROS-mediated activation of the AMPK/mTOR signaling pathway.

This Erastin-induced increase in autophagic flux is crucial because it directly enables ferritinophagy, a selective process that targets the iron-storage protein ferritin for degradation and is now recognized as a key driver of ferroptosis. Ferritinophagy is the autophagic degradation process of ferritin, an iron storage protein, which is crucial for cellular iron homeostasis. Ferritin consists of 24 subunits of ferritin heavy chain 1 (FTH1) and ferritin light chain (FTL), capable of storing up to 4,500 iron atoms (51). The combination of autophagosome isolation and quantitative proteomics has identified nuclear receptor coactivator 4 (NCOA4) as the cargo receptor responsible for autophagy-dependent ferritin degradation (52). This mechanism is likely due to the C-terminal domain of NCOA4 binding to the conserved surface arginine (R23) of FTH1 in phagocytic cells, subsequently facilitating its interaction with autophagosomes and autolysosomes (14). Mancias et al. demonstrated that ferritin delivery to lysosomes requires NCOA4, and NCOA4-deficient cells fail to degrade ferritin, leading to a reduction in bioavailable intracellular iron (53). Supporting this direct link, Hou et al. identified NCOA4 as the selective cargo receptor for the autophagic turnover of ferritin during ferroptosis, demonstrating that genetic inhibition of NCOA4 suppressed ferritin degradation and consequently inhibited Erastin-induced ferroptosis (54). In line with this, Sun et al. noted that the knockdown of NCOA4 or core autophagy genes like ATG5 and ATG13 could suppress Erastin-induced ferritin degradation, iron accumulation, and lipid peroxidation. Therefore, by activating this NCOA4-mediated ferritinophagy pathway, Erastin promotes the release of free iron, which in turn amplifies ROS production and lipid peroxidation, resulting in an autophagy-dependent mode of ferroptosis. Given this evidence, it is clear that Erastin-induced cell death in EMS tissue involves a critical autophagic component, wherein the induction of NCOA4-mediated ferritinophagy drives ferroptosis by degrading ferritin and increasing the intracellular labile iron pool.

## 6 Mitophagy and mitochondrial quality control in Erastin sensitivity

Within the broader process of autophagy, mitophagy—the selective removal of mitochondria—plays a particularly intricate role in the response of endometriotic cells to Erastin. Mitophagy is a highly selective form of autophagy responsible for eliminating damaged mitochondria, thereby maintaining mitochondrial quality control and cellular homeostasis (55). This process is canonically driven by the stabilization of PTEN-induced kinase 1 (PINK1) on the outer membrane of depolarized mitochondria, which subsequently recruits the E3 ubiquitin ligase Parkin (56). Parkin-mediated polyubiquitination then facilitates the interaction with autophagy receptors, ultimately leading to autophagosome formation and lysosomal degradation of dysfunctional mitochondria. In the context of EMS, impaired mitophagy has significant pathological implications, resulting in the accumulation of dysfunctional mitochondria and elevated levels of ROS. Providing direct molecular evidence for this impairment, Deng et al. identified Prohibitin 2 (PHB2), a critical mitochondrial inner-membrane receptor for Parkin-dependent mitophagy, as significantly downregulated in ectopic endometrial tissues (57). However, the clinical persistence of EMS, a disease state characterized by impaired mitophagy, suggests that the resulting elevation in ROS is not sufficient to induce widespread EESC death. If it were, EMS would likely behave as a self-limiting condition. Instead, this limited increase in ROS appears to place EESCs in a state of chronic oxidative stress, which fuels a pro-inflammatory and pro-proliferative microenvironment rather than effectively killing the ectopic cells.

This chronic oxidative stress state, and the pro-inflammatory, pro-proliferative microenvironment it creates, can be controlled through the intervention of restoring mitophagy. Evidence from animal models demonstrates that reactivating mitophagy effectively limits the inflammatory microenvironment, reduces angiogenesis, and promotes the clearance of ectopic cells. In a rat model of EMS, D’Amico et al. demonstrated that supplementation with açai berry suppresses the PI3K/AKT and ERK signaling pathways, thereby inhibiting mammalian target of mTOR activity and activating AMBRA1/Beclin-1–LC3 signaling (58). This cascade restores PINK1/Parkin-dependent mitophagy and concurrently activates the NRF2 pathway, enhancing antioxidant responses, like NQO-1and HO-1, and consequently reducing oxidative stress in ectopic lesions. Complementing this finding, Siracusa et al. reported that ectopic endometriotic implants show reduced expression of key mitophagy proteins, including BNIP3, AMBRA1, and Parkin, which further confirms that mitophagy is suppressed in EESCs (59). Treatment with rapamycin reversed these impairments by downregulating the phosphorylated AKT/mTOR pathway and upregulating BNIP3, AMBRA1, and Parkin expression. Crucially, their studies found that the restoration of mitophagy leads to increased apoptosis, decreased angiogenesis, and notably smaller lesion sizes. Deng et al. show that overexpression of PHB2—as a key receptor for mitophagy, its overexpression signifies an effective restoration of this process—suppressed cellular proliferation, migration, and invasion, and promoted apoptosis through the enhancement of Parkin-dependent mitophagy (57). This regulation was shown to occur under the direct transcriptional control of GA-binding protein alpha (GABPA)—a critical Ets-family transcription factor that acts as a master regulator of mitochondrial biogenesis by controlling the expression of nuclear-encoded mitochondrial proteins, and whose direct binding to the PHB2 promoter makes it a key upstream determinant of the cell’s capacity for mitophagy (60).

Additional mechanistic insights have further delineated the complexity of mitophagy regulation in EMS. In parallel, Zhao et al. illustrated another regulatory axis wherein mammalian Ste20-like kinase 1 (MST1), which is downregulated in endometriosis, modulates mitochondrial dynamics. Their work showed that restoring MST1 expression activates p53, which subsequently phosphorylates Drp1 at Ser616 to facilitate mitochondrial fission while concurrently suppresses Parkin transcription (61). While this pathway also impairs mitophagy, it does so to an extent that pushes the cell past a survival threshold, ultimately inducing caspase-9-dependent apoptosis and loss of migratory capacity in EESCs. This highlights that EESCs exist in a delicate balance, where their survival depends on a moderate level of mitochondrial dysfunction; restoring homeostasis through conventional mitophagy or inducing catastrophic mitochondrial failure can both lead to their demise.

Recent investigations have uncovered a direct mechanistic interplay between mitophagy and Erastin-induced ferroptosis in EMS. Gou et al. found that butyrate, a short-chain fatty acid derived from gut microbiota—whose levels are notably diminished in EMS patients—heightens the susceptibility of EESCs to Erastin-induced ferroptosis (62). This sensitization effect occurs via the suppression of mitophagy through the FFAR2/PPAR-*γ*/PINK1/Parkin axis. Knockdown or pharmacological inhibition of components within this axis effectively reversed the ferroptosis-enhancing impact of butyrate, thus establishing a functional and causal relationship between mitophagy suppression and heightened Erastin responsiveness. The mTOR pathway, serving as a critical metabolic regulator and mitophagy gatekeeper, integrates both metabolic and inflammatory signals to calibrate mitochondrial turnover and ROS production—key mediators of ferroptosis induction by Erastin (63–65). Under pathological conditions characterized by deficient mitophagy, pharmacological agents such as rapamycin or açai berry restore mitochondrial turnover, thereby promoting apoptosis and reducing angiogenesis in lesions reliant on AKT/mTOR signaling (66–68). Conversely, in cellular contexts dependent upon Parkin-mediated mitophagy for survival, interference with this pathway via MST1-p53 axis activation or FFAR2/PPAR-*γ* signaling blockade enhances cellular susceptibility to ferroptosis upon Erastin treatment.

Collectively, current evidence highlights a nuanced and context-dependent role for mitophagy in modulating the therapeutic efficacy of Erastin in EMS. Under physiological or untreated pathological conditions, mitophagy functions beneficially by maintaining mitochondrial integrity, reducing ROS accumulation, suppressing oxidative stress, and consequently decreasing angiogenesis and lesion viability in EESCs. However, EMS lesions inherently exhibit impaired mitophagy, leading to the persistent accumulation of dysfunctional mitochondria and increased ROS production. Crucially, this dysfunctional state provides a unique opportunity for Erastin-based therapy. Erastin exploits the defective mitophagy-driven ROS accumulation to circumvent the ferroptosis resistance characteristic of EESCs, thereby enhancing its therapeutic efficacy. Conversely, the restoration or enhancement of mitophagy would paradoxically attenuate Erastin-induced ROS accumulation, undermining its ferroptosis-promoting effects. Therefore, to maximize the therapeutic potential of Erastin, targeted suppression of mitophagy during treatment is essential. Future research should further explore this critical balance, aiming to define precise therapeutic windows and identify molecular targets within mitophagy pathways to optimize Erastin’s clinical application in treating endometriosis.

## 7 Erastin-induced multi-pathway cell death network in EMS

In EMS tissue, Erastin activates multiple cell death signaling pathways, forming a complex network effect that ultimately induces the death of ectopic endometrial cells. First, by inhibiting system Xc^−^, Erastin causes a drastic reduction in GSH levels, leading to the inactivation of GPX4 and the accumulation of lipid peroxides, thus inducing and amplifying ferroptosis. This process is closely associated with autophagy, particularly with NCOA4-mediated ferritinophagy. In the case of high autophagic flux, ferritin is degraded, releasing more Fe^2+^, which further promotes the Fenton reaction and lipid peroxidation, thereby exacerbating ferroptosis and causing greater membrane damage. In the context of FPN deficiency or inadequate expression, the cells cannot efficiently export iron, resulting in continued accumulation of free iron and further enhancing the detrimental effects of ferroptosis.

It is important to note that Erastin-induced cell death is not limited to ferroptosis. Following the generation of large amounts of ROS, necroptosis is activated. Necroptosis, driven by the RIPK1–RIPK3–MLKL signaling cascade, is amplified in the presence of ROS, leading to the formation of membrane pores and membrane rupture, which releases inflammatory molecules and additional ROS. This not only exacerbates the damage to ectopic endometrial cells but also creates a sustained oxidative stress and inflammatory signal in the local microenvironment. Meanwhile, pyroptosis can also be indirectly triggered by Erastin. As ROS accumulate excessively, the mitochondrial outer membrane protein Tom20 and the Bax pathway are altered, leading to the release of cytochrome c and other pro-apoptotic/pro-pyroptotic molecules from mitochondria, thereby activating the downstream caspase cascade. The cleaved N-terminal fragment of GSDME forms pores in the cell membrane, triggering pyroptosis, which is characterized by cell swelling, rupture, and the release of pro-inflammatory signals. As the damage to the membrane structure and organelles increases, a new wave of ROS and inflammatory factors further feedback into the pathways of ferroptosis and necroptosis, amplifying their effects.

Notably, the unique pathological context of EMS critically shapes the outcome of these interconnected pathways. While autophagy is normally a protective mechanism, Erastin subverts this process to promote ferritinophagy, leading to the large-scale degradation of ferritin and the release of excess iron that further reinforces ferroptosis. More specifically, mitophagy, the programmed quality-control pathway for mitochondria, becomes maladaptive in the EMS context. The defective mitophagy observed in EESCs permits the accumulation of dysfunctional mitochondria, which sustains elevated ROS levels and intensifies all ROS-dependent arms of Erastin-induced cell death. Taken together, the convergence of ferritinophagy-derived labile iron and pathology-related mitophagy deficiency creates a hypersensitive state, heightening the induction of cell death by Erastin in EESCs. On this basis, we posit that the therapeutic action of Erastin in EMS is critically mediated by these ROS-driven programs, and that pharmacologic suppression of mitophagy may further sensitize lesions to Erastin.

**Figure 4:**
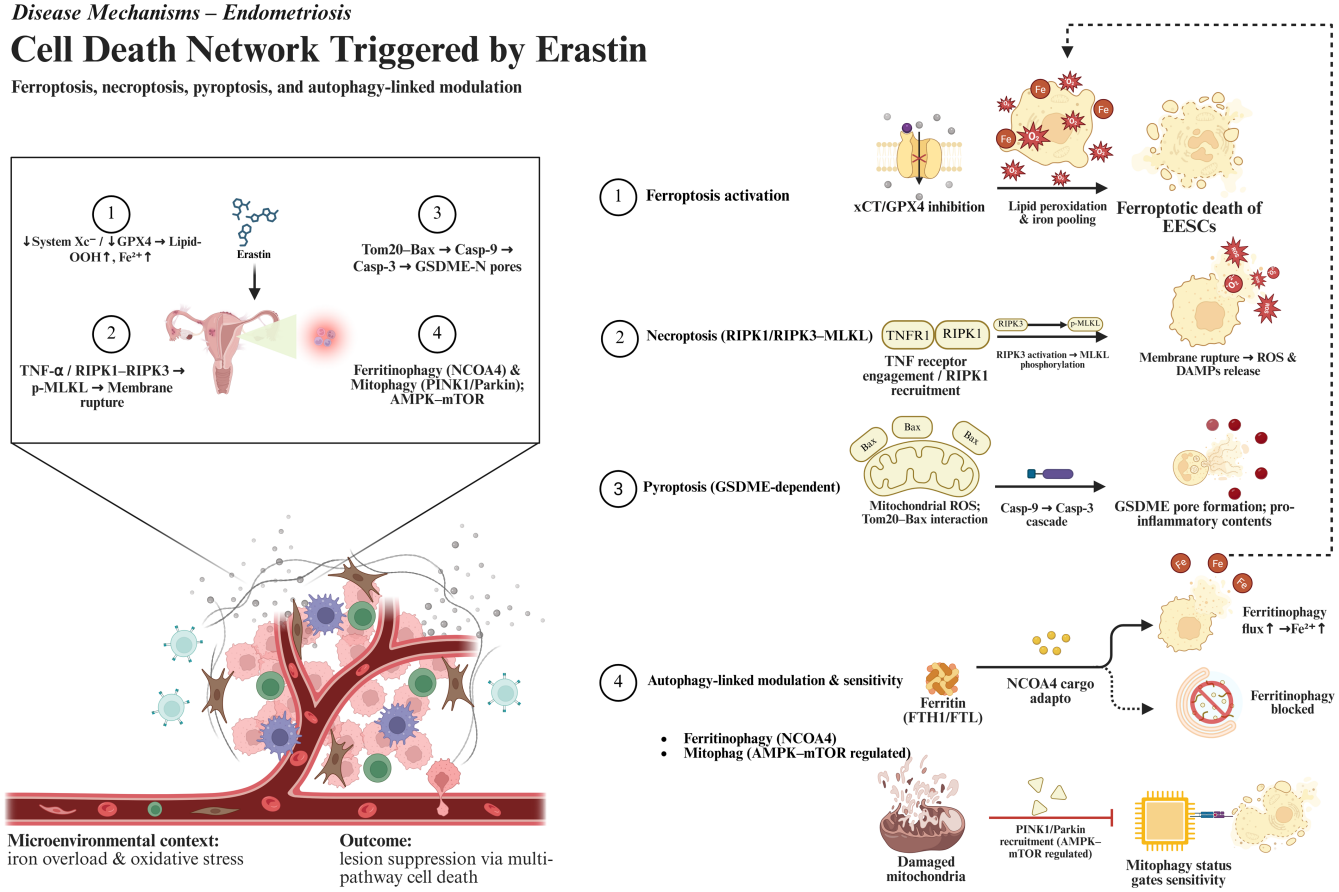
The multi-pathway regulated cell death network induced by Erastin in endometriosis. This network is centered around free iron and ROS, which are amplified through interconnected cell death programs. The figure details the key pathways, including: (1) ferroptosis activation through system Xc^−^/GPX4 inhibition, (2) necroptosis triggered by the RIPK1-RIPK3-MLKL signaling cascade, (3) pyroptosis mediated by the Tom20-Bax-Caspase-3/GSDME axis, and (4) the crucial modulation of these pathways by ferritinophagy, which increases labile iron, and defective mitophagy, which sustains elevated ROS levels.

Based on these findings, we propose that the multiple cell death pathways induced by Erastin in EMS create a tightly regulated positive feedback network centered around free iron and ROS. Figure 4 shows the integrated network of cell death pathways triggered by Erastin in endometriosis, illustrating how ferroptosis, necroptosis, and pyroptosis are interconnected and modulated by autophagy-dependent processes. On one hand, free iron and ROS are the core driving factors of ferroptosis; on the other hand, ROS explosion and the spread of inflammatory signals further enhance necroptosis and pyroptosis, and in some cases, affect autophagic function. These death modes interact with and amplify each other, leading to the rapid and compounded destruction of ectopic endometrial tissue. By precisely regulating key molecules involved in iron homeostasis and autophagy, such as FPN, heme oxygenase, and NCOA4, it may be possible to improve the specificity and effectiveness of Erastin in EMS treatment, while minimizing excessive inflammation or oxidative damage to surrounding normal tissues. Further research into these feedback loops and critical molecular nodes may uncover more potential small molecules or biomarkers, providing new insights for the clinical translation of Erastin-based therapies in EMS and potentially other diseases.

## 8 Translational outlook: analogs, safety liabilities, and delivery strategies

Erastin has demonstrated antitumor activity in several malignancies, including gastric and colorectal cancer (69, 70), and the inflammatory, iron-loaded, oxidative microenvironment shared by many tumors and EMS provides a coherent theoretical basis for applying this strategy to EMS (71, 72). Building on the evidence of Erastin-induced cell death mentioned earlier, we posit that Erastin can restrain lesion growth in EMS by inhibiting system Xc^−^, depleting cystine and GSH, compromising GPX4 activity, and driving lipid peroxidation within EESCs. Under the iron-rich, ROS-prone conditions characteristic of lesions, this perturbation engages a coordinated program of RCD across intersecting pathways—not only ferroptosis but also necroptotic and pyroptotic signaling—while ferritin handling and mitophagy status further skew redox homeostasis toward irreversible injury in EESCs. Studies have shown that EESCs are more vulnerable than NESCs in this context, and that the lesion milieu amplifies Erastin’s impact through persistent oxidative pressure and impaired mitochondrial quality control (26, 38, 73, 74). Taken together with precedent from oncology, the overall evidence indicates that Erastin has clear therapeutic potential for EMS, provided drug delivery preferentially targets lesions while sparing healthy tissue.

Medicinal chemistry efforts have generated analogs and functional derivatives to address Erastin’s poor solubility, metabolic instability, and limited usability *in vivo* (75). Imidazole ketone Erastin (IKE) achieves markedly greater potency and stability through an imidazole ketone scaffold that enables durable target engagement while resisting metabolic clearance (76); in cell assays it reaches nanomolar activity and maintains stability in plasma and liver microsomes, and in animal models it inhibits tumor growth at tolerated doses with suitable formulations (77). Piperazine Erastin (PE) increases polarity and water compatibility, raising aqueous solubility by more than an order of magnitude relative to Erastin and improving exposure and metabolic stability (78, 79); despite only modest gains in intrinsic potency, it achieves effective concentrations *in vivo* and delays tumor growth in xenograft models (12). PRLX93936 was optimized for drug like behavior and formulated as a hydrochloride salt to enhance bioavailability; it demonstrated preclinical efficacy (80), progressed to first in human testing, and showed activity in Ras driven models, including combinational benefit with platinum therapy (81, 82). Sulfasalazine (SAS) is an orally available, water soluble inhibitor of system Xc^−^ with a well characterized clinical pharmacology (83); although less potent than Erastin *in vitro*, its feasibility for systemic dosing and observed ferroptosis linked sensitization in difficult models underscore its translational relevance (84). RSL3 and ML162 represent a second strategy that disables lipid peroxide repair by directly inhibiting GPX4 with low nanomolar lethality in diverse cell systems; their electrophilic chemistry and rapid reactivity limit systemic use at present but they define a potent downstream entry point for ferroptosis (85, 86). FIN56 promotes GPX4 degradation while depleting coenzyme Q10, creating a dual node pressure on the lipid antioxidant network that can overcome single target resistance and broaden ferroptosis induction across resistant states (87, 88).

**Figure 5:**
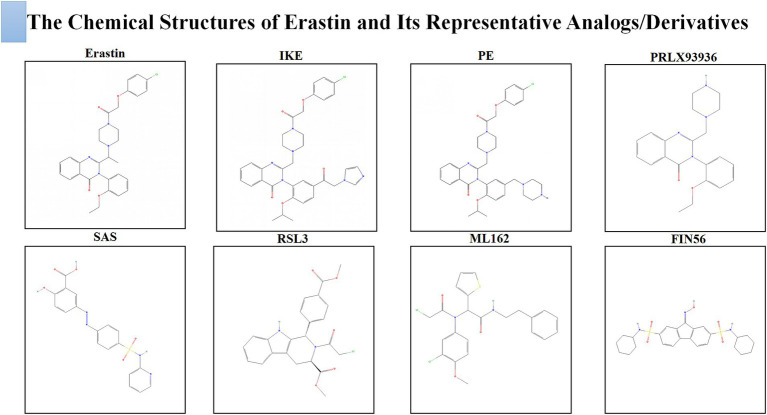
The chemical structures of Erastin and its representative analogs/derivatives.

Across these compounds, two mechanistic classes emerge. The first starves cells of cystine by inhibiting system Xc^−^, collapsing the GSH–GPX4 axis and predisposing lesions to lipid peroxidation; this class includes Erastin, IKE, PE, PRLX93936, and SAS. The second disrupts lipid peroxide repair at or downstream of GPX4 through direct enzyme blockade or by accelerating GPX4 loss and depleting its cofactors; this class includes RSL3, ML162, and FIN56. Figure 5 shows the chemical structures of Erastin and these representative analogs/derivatives. Table 1 shows the pharmacological properties and practical advantages of these agents relative to Erastin, providing a reference framework for selecting candidates and delivery strategies in preclinical models of EMS.

**Table 1:** Pharmacological characteristics and advantages compared to Erastin for representative analogs/derivatives.

Compound	Main pharmacological characteristics	Advantages compared to Erastin
Erastin	A system Xc^−^ inhibitor with poor aqueous solubility and metabolic instability.	Mechanism benchmark for ferroptosis in EMS-relevant settings.
IKE	A system Xc^−^ inhibitor with nanomolar potency and improved metabolic stability.	Delivers stronger activity at lower doses with improved metabolic robustness.
PE	A system Xc^−^ inhibitor with greatly increased aqueous solubility and better systemic exposure.	Enables simple aqueous formulation and higher systemic exposure.
PRLX93936	A system Xc^−^ inhibitor formulated as a salt with drug-like pharmacokinetics and human data.	Offers translational pharmacokinetics with first-in-human experience.
SAS	An oral, water-soluble system Xc^−^ inhibitor suitable for systemic dosing.	Provides a clinically approved, systemically dosable scaffold for repurposing.
RSL3	A direct GPX4 inhibitor that produces rapid, potent ferroptosis.	Bypasses cystine dependence through direct GPX4 blockade.
ML162	A direct GPX4 inhibitor used as a potent tool compound in resistant models.	Affords robust GPX4 inhibition for models resistant to upstream blockade.
FIN56	An agent that promotes GPX4 degradation and depletes coenzyme Q10 to drive ferroptosis.	Exerts dual pressure on the lipid-antioxidant network to overcome resistance.

Despite rapid advances in the design of Erastin analogs and derivatives, the fundamental problem of on-target toxicity remains unresolved. Inhibiting system Xc^−^ lowers intracellular cystine and depletes GSH in transporter-dependent tissues, placing normal cells under sustained oxidative pressure (89). Studies have shown in mouse models that high-dose Erastin reduces red blood cells and hemoglobin, suppresses bone-marrow cellularity, and depletes hematopoietic stem and progenitor populations that are highly sensitive to ferroptosis (90, 91); immune cell death has also been observed, weakening antitumor immunity (92). Consistent with transporter dependence in the gastrointestinal tract, kidney, liver, and spleen (89), tissue injury emerges when systemic exposures are achieved. Agents that act downstream on GPX4 carry a related liability because they disable lipid-peroxide repair in nonlesional tissues. Next-generation compounds improve chemistry rather than biology: IKE increases potency and stability and PE increases aqueous solubility and exposure, yet both remain system Xc^−^ inhibitors and therefore retain the same on-target risk. Clinical experience with PRLX93936 underscores the ceiling for systemic dosing, as a phase I trial was terminated early when patients experienced severe, intolerable toxicity at very low doses (93), revealing an exceptionally narrow therapeutic index.

Beyond on-target injury, off-target interactions further complicate translation. Mitochondrial engagement, including binding to voltage-dependent anion channels, can heighten oxidative stress in nonlesional tissues and contribute to organ dysfunction independent of lesion-directed ferroptosis (94, 95). Studies have shown that Erastin can also modulate p53 signaling outside its canonical ferroptosis activity, altering transcriptional programs that regulate cell-cycle progression and stress responses and thereby intensifying cytotoxicity in sensitive tissues (96). Taken together, the combined burden of on-target toxicity and off-target effects constrains the therapeutic window and constitutes a practical barrier to clinical development, making it essential to pair analog optimization with delivery strategies that confine exposure to lesions and minimize unintended pathway engagement in normal tissues.

In light of the on-target and off-target liabilities outlined above, we recommend a development path that prioritizes separation of lesion efficacy from systemic exposure. The first layer should focus on biodistribution: local or lesion-preferring delivery is likely to yield the greatest gain in therapeutic index. We propose testing intralesional or intraperitoneal depot systems, as well as nanoparticle and liposomal carriers engineered to concentrate drug within pelvic lesions while limiting distribution to hematopoietic and hepatic tissues (97–99). As a complementary approach, rational prodrug designs and receptor-targeted conjugates should be explored to restrict activation to cells that overexpress disease-associated markers in the endometriotic microenvironment. Triggerable chemistries responsive to local redox state, pH, or lesion-enriched enzymes may further ensure that active drug is generated predominantly within ectopic tissue (100). Together, these delivery-centered strategies provide the mechanistic context needed to revisit exposure levels that were previously precluded by toxicity.

We also recommend schedule and combination designs that maintain lesion pressure while protecting normal tissues. Intermittent or metronomic dosing could preserve antioxidant capacity in nonlesional compartments yet sustain cumulative oxidative stress in EESCs (89). Combination regimens that sensitize lesions—by increasing iron availability within lesions or dampening compensatory antioxidant pathways—may allow meaningful dose reductions of Erastin-class agents. To enable safe clinical exploration, pharmacodynamic monitoring should be embedded from the outset, using biomarkers of lipid peroxidation and cellular redox status to verify on-lesion activity, coupled with early indicators of marrow and mitochondrial injury to guide dose adjustment (101). Patient selection should be biomarker-guided, emphasizing lesions with molecular features consistent with ferroptosis susceptibility and delivery feasibility (102). Finally, medicinal chemistry should advance analogs with improved stability and minimized promiscuous protein binding, specifically aiming to reduce marrow suppression and mitochondrial liabilities while retaining on-lesion potency (103).

Taken together, current evidence positions Erastin and its derivatives as plausible candidates for controlling endometriosis by exploiting the iron-driven, oxidative lesion milieu to trigger RCD in ectopic stromal cells. Two complementary lineages—system Xc^−^ inhibitors and GPX4-directed agents—offer improvements in potency, stability, or solubility, yet translation is constrained by on-target depletion of cystine and glutathione in normal tissues and by off-target perturbations, including mitochondrial channel engagement and p53 pathway modulation, which collectively narrow the therapeutic index. Progress will depend on decoupling lesion efficacy from systemic exposure through lesion-focused delivery platforms, triggerable or receptor-targeted prodrugs, and schedules or combinations that sustain lesion pressure at reduced doses. Pharmacodynamic monitoring and biomarker-guided selection should verify on-lesion activity while safeguarding marrow and mitochondrial function. Priorities for research include head-to-head evaluation of analogs in EMS models with integrated pharmacokinetic–pharmacodynamic mapping and iterative chemistry to lower promiscuous binding without compromising on-lesion potency.

## 9 Conclusions and future directions

Evidence synthesized in this review indicates that Erastin has potential to control EMS by exploiting lesion-specific vulnerabilities in iron handling and redox balance. Within the iron-rich, ROS-prone microenvironment of EMS, inhibition of system Xc^−^ limits cystine import, depletes GSH, constrains GPX4 activity, and drives lipid peroxidation in ectopic endometrial stromal cells. Under these conditions, Erastin engages a coordinated network of regulated cell death that extends beyond ferroptosis to include necroptotic and pyroptotic signaling, with ferritin turnover increasing labile iron and mitophagy status shaping cellular susceptibility. Studies across models, together with precedent from oncology, support the view that this multi-pathway engagement can be leveraged to restrain lesion growth when exposure is directed preferentially to diseased tissue.

Translation is limited at present by on-target injury in normal tissues and by off-target pathway perturbations, necessitating solutions that separate lesion efficacy from systemic risk. Future work should prioritize delivery platforms that localize exposure to pelvic lesions, including intraperitoneal or intralesional depots and nanocarriers, alongside triggerable or receptor-guided prodrugs designed to activate within the biochemical milieu of lesions. Scheduling and combination strategies that maintain pressure on ectopic cells while preserving antioxidant capacity in nonlesional compartments warrant systematic evaluation, supported by pharmacokinetic–pharmacodynamic mapping and biomarker programs that verify on-lesion lipid peroxidation and redox modulation while monitoring marrow, hepatic, renal, and mitochondrial safety. Comparative studies of representative analogs under uniform conditions, coupled with iterative chemistry to minimize promiscuous protein binding and mitigate p53- and mitochondrial channel–linked liabilities, will be essential. Attention to reproductive endpoints and long-term outcomes should anchor this agenda so that Erastin-class approaches can be tested credibly for clinical benefit in EMS.
